# The Non-Coding RNA Landscape in IgA Nephropathy—Where Are We in 2021?

**DOI:** 10.3390/jcm10112369

**Published:** 2021-05-28

**Authors:** Izabella Z. A. Pawluczyk, Haresh Selvaskandan, Jonathan Barratt

**Affiliations:** Department of Cardiovascular Sciences, University of Leicester, Leicester LE1 7RH, UK; hs328@le.ac.uk (H.S.); jb81@le.ac.uk (J.B.)

**Keywords:** IgA nephropathy, RNA interference, microRNA

## Abstract

IgA nephropathy (IgAN) is the most commonly diagnosed primary glomerulonephritis worldwide. It is a slow progressing disease with approximately 30% of cases reaching end-stage kidney disease within 20 years of diagnosis. It is currently only diagnosed by an invasive biopsy and treatment options are limited. However, the current surge in interest in RNA interference is opening up new horizons for the use of this new technology in the field of IgAN management. A greater understanding of the fundamentals of RNA interference offers exciting possibilities both for biomarker discovery and, more importantly, for novel therapeutic approaches to target key pathogenic pathways in IgAN. This review aims to summarise the RNA interference literature in the context of microRNAs and their association with the multifaceted aspects of IgA nephropathy.

## 1. Non-Coding RNA

For many years, most RNA research focused on messenger RNAs (mRNA) as the key intermediaries in the translation of DNA gene sequences into proteins, and apart from ribosomal (rRNA) and transfer RNAs (tRNA), which play central roles in protein synthesis, other ‘non-coding’ RNAs were overlooked as by-products of transcription. However, since the advent of high throughput sequencing technologies and bioinformatics analyses, numerous types of non-coding RNAs (ncRNA) have been discovered that are now recognised as playing fundamental roles in a broad spectrum of cellular processes. These ncRNAs can be divided into two groups, although there may be some overlap: those that perform a housekeeping function and those that play regulatory roles (Table 1) [1] (see also [2] for a more exhaustive list). House-keeping RNAs are constitutively and abundantly expressed in all cells and are essential for cell viability [3]. Regulatory ncRNAs are involved in gene expression as well as genome maintenance and regulation [reviewed in 1]. Non-coding RNAs can be found in both short (<30 nucleotides) and long forms (>200 nucleotides). The short forms are subdivided into three major classes; microRNAs (miRs), the most abundant class, are transcribed from the non-coding regions of the genome [4], short interfering RNAs (siRNA) are processed from exogenous viral or artificially inserted DNA [5] as well as from endogenous transposon elements [6], while PIWI (P-element Induced WImpy testis in Drosophila)-interacting RNAs (piRNA) are processed from single stranded RNA precursors and only function through PIWI protein binding [1]. Long noncoding RNAs are usually longer than 200 nucleotides and are further subdivided into five categories depending on whether they are transcribed from the sense or antisense DNA strand and within which region of the genome (intron, exon, intergenic or overlapping regions) [7].

## 2. RNA Interference

Investigations in the late 1970s using single stranded oligonucleotide antisense therapy to manipulate gene expression at best delivered modest results [8], but serendipitously led to the discovery that double stranded ncRNAs had potent suppressive effects on mRNA transcription [9]. This breakthrough launched a revolutionary new age of RNA interference (RNAi) which has transformed basic biological research as well as translational medicine. Five years earlier, in 1993, transcriptional silencing by microRNAs (miRs) binding to the 3’UTR of target genes was first reported [4]. Extensive research eventually unified the RNAi and miR pathways when it was discovered that they shared core mechanistic components [10].

**Figure 1:**
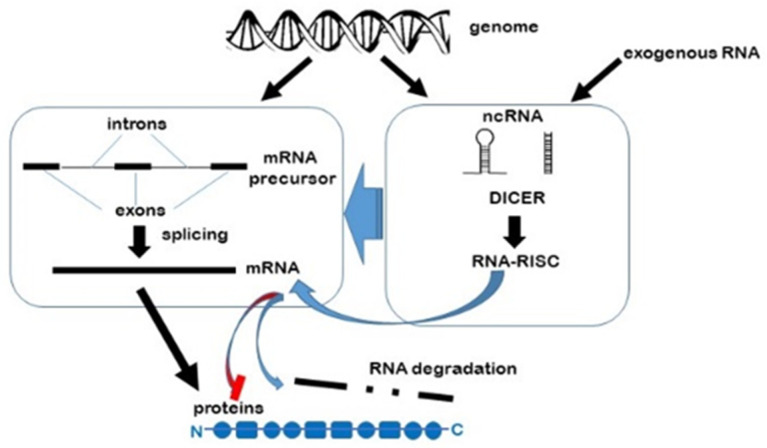
RNA interference. The influence non-coding RNAs have on mRNA once it has been transcribed from its gene on its journey to the ribosome for translation into protein.

RNAi pathways are naturally found in all living organisms [11], regulating and fine tuning gene transcription and post transcriptional processes, as well contributing to genome organisation and stability (Figure 1). However, the fact that synthetic double stranded ncRNAs can be introduced into cultured cells as well as whole living organisms to selectively suppress transcription has made them invaluable tools in basic biology research, biotechnology and medicine. 

## 3. MicroRNAs

MicroRNAs (miRs) are small ncRNAs which negatively regulate gene expression, fine tuning physiological processes involved in proliferation, apoptosis, differentiation and development [4,12]. Dysregulated miR expression has been associated with the pathophysiology of a variety of diseases, including heart disease, cancer, diabetes mellitus and kidney disease [13,14,15]. 

At the time of writing, there were 3797 citations identified using the search terms ‘microRNA’ and ‘kidney’ in PubMed (United States National Library of Medicine at the National Institutes of Health), with two thirds of these having been published in the last 4 years. A recognition that miRs influence the function of all segments of the nephron and that dysregulation occurs at all stages of kidney disease from disease onset to the final common pathways of CKD progression explains the intense interest in studying miRs as minimally invasive biomarkers and novel therapeutic targets. This review will focus on the potential role of miRs in the pathogenesis of IgAN, the biomarker discovery studies reporting miR perturbations in IgAN and the new studies using RNA interference strategies to treat IgAN.

### MiR Biogenesis

**Figure 2:**
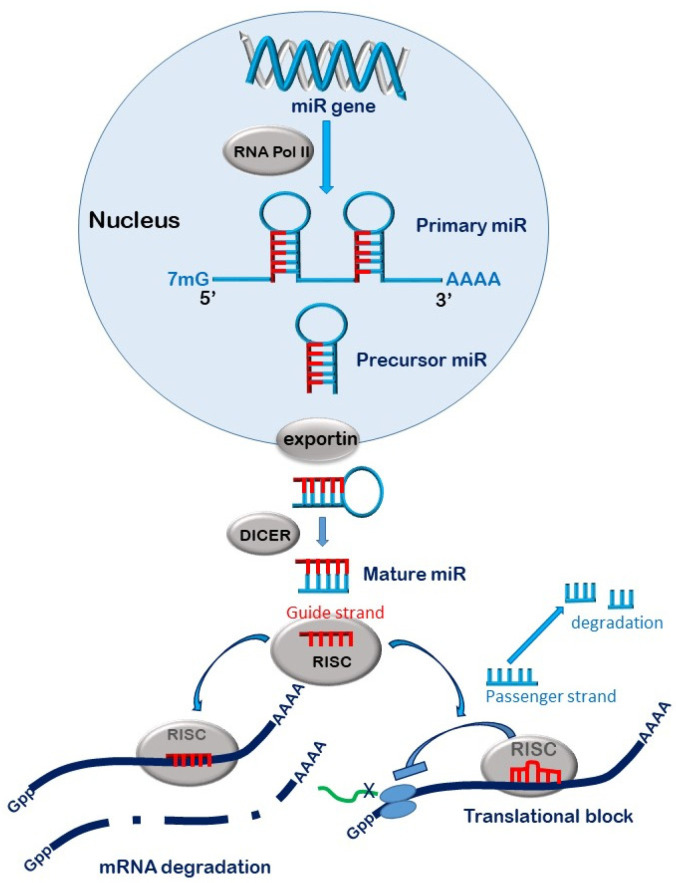
Biogenesis of microRNAs. MicroRNAs are transcribed from the non-coding regions of the genome by the actions of RNA polymerase 2. The resultant hairpin-looped primary miR (pri-miR) undergoes post-transcriptional editing by the actions of the RNAse Drosha, supported by the action of DGCR8 which cleaves the pri-miR at its 5’ and 3’ regions to produce a precursor miR (pre-miR). The pre-miR is exported out of the nucleus by the Exportin 5-Ran-GTP complex, which also protects it from degradation. The pre-miR undergoes further editing by the actions of Dicer, which cleaves off the hairpin loop structure to generate short double stranded microRNA. The two miR strands are separated by helicases. The less thermodynamically stable ‘guide’ strand is loaded onto the argonaute protein within the RNA-induced silencing complex (RISC). The remaining ‘passenger’ strand is cleaved and degraded in the cytoplasm. The RISC is then guided to its target site on mRNA where, depending on complementarity, it will degrade the mRNA or disrupt its translation.

The accepted canonical dogma is that miRs are transcribed from genes or ‘transcription sites’ in the nucleus by RNA polymerases (II and III) to form primary miR (pri-miR) transcripts several kilo bases in length and exhibiting a unique hairpin loop within their structure. The 5’ and 3’ ends of the pri-miRs are trimmed by the action of the RNAse III endonuclease Drosha complexed to its cleavage partner DCGCR 8 (DiGeorge Syndrome Critical Region 8) to form 70–100 nucleotide precursor-microRNAs (pre-miRs) [16]. The pre-miRs shuttle out of the nucleus by coupling to exportin 5 complexed to GTPase RAN [17]. As the complex traverses through the nuclear pore, the GTP is hydrolysed to GDP, releasing the pre-miR into the cytoplasmic compartment [18]. Here, the pre-miRs undergo further processing by the actions of the double stranded RNA-specific RNAse III endonuclease Dicer, which removes the hairpin loop structure to form a linear double stranded RNA [19]. This RNA duplex is loaded onto the argonaute (AGO) proteins within the RNA-induced silencing complex (RISC) [20]. The less thermodynamically stable of the two strands remains on the AGO as the functional ‘guide’ strand, while the ‘passenger’ stand is expelled from the complex and degraded [21]. The guide strand then directs the mature RISC complex to its target site on the 3’ untranslated (3’UTR) region of mRNA. There are four AGOs in humans all capable of binding and silencing miRs, although only AGO-2 possesses endonuclease activity capable of cleaving target mRNA [22]. The effect of the miR on the target mRNA will depend on the degree of complementarity between the 7 base ‘seed’ sequence (bases 2–8) of the mature miR with its target region in the mRNA [23]. Total complementarity will result in cleavage of the mRNA transcript by the AGO-2, followed by complete degradation by cytoplasmic exoribonuleases [21] (Figure 2). Partial complementarity, which occurs in most cases, will result in the inhibition of protein translation [24,25]. Although miRs largely target areas of the 3’UTR region of their target mRNA, some can bind to the 5’ UTR and/or the coding region, resulting in similar outcomes [26]. It is noteworthy that while miR silencing is said to occur in the cytosol, it principally takes place on the membranes of endoplasmic reticulum and can also occur within cellular organelles such as the mitochondria, the nucleus and nucleolus [27]. 

Approximately 52% of human miR ‘transcription sites’ are located in the intergenic regions of DNA, while 40% lie within intronic regions and 8% are exonic [28]. MiRs within introns (or exons) that are orientated in a ‘sense’ direction with the coding region are transcribed as part of the host gene [29], while miRs that are encoded in the intergenic regions are transcribed as independent ‘transcription units’ having their own promoter apparatus [30,31,32,33]. Similarly, miRs that are derived from introns and/or exons in the antisense orientation are also independently transcribed [33]. 

The half-life of miRs can depend on prevailing conditions; often it is quite long with miRs persisting for some 5 days or longer, but some are turned over very rapidly [34]. A number of miRs are conserved between organisms, suggesting they control essential processes [35,36]. However, the function of many miRs remains to be determined. Some miRs can downregulate large numbers of targets [37], making it a challenge to identify the functional role of any one miR on any single target. However, some miRs do exhibit significant tissue specificity. For example, miR-122 is highly associated with the liver [38], miRs-124 and -134 with the brain [39], and miRs-1 and -133 with muscle [40]. 

## 4. IgA Nephropathy

While the pathogenesis of IgAN is still incompletely understood, it is widely considered to involve four major events (‘four hit hypothesis’ [41]), which result in immune complexes of poorly *O*-galactosylated IgA1 depositing within the glomerular mesangium and initiating local inflammatory reactions, the sequelae of which lead to kidney damage, and in some 30% of patients, end-stage kidney disease. Given the ubiquitous nature of their role in health and disease, dysregulated miR expression is highly likely to play a role in the multifaceted disease that is IgAN. 

## 5. MiRs in IgA Nephropathy 

### 5.1. Kidney Tissue and Cultured Kidney Cells

MiR analyses in both human and mouse tissue have indicated that a small group of miRs (-192, -194, -204, -215 and -216) are enriched in the kidney compared to other organ systems [42]. Moreover, seven miRs, -30d, -140-3p, 532-3p, -194, -190, -204, and -206, have been shown to be significantly downregulated in progressive forms of kidney disease [43]. The expression of these miRs inversely correlates with the expression of 29 target mRNAs shown by pathway analysis to be involved in the inflammatory response, cell–cell interaction, apoptosis and intracellular signalling [43].

In a study using microarray analysis, Dai et al. [44] first demonstrated that in IgAN 34 miRs were upregulated and 31 downregulated compared to healthy subjects. Tan et al. [45] performed similar investigations using high throughput RNA sequencing and reported that 84 miRs were differentially expressed between IgAN and healthy subjects. Unfortunately, both studies were small and neither included non-IgAN kidney disease controls or validated their data by RT-PCR in independent sets of cohorts. Other intra-kidney IgAN-based studies [46,47,48,49] have reported that miRs -21-5p, -155, -199a-5p, -205, -214-3p have each been shown to correlate with the extent of interstitial fibrosis, a powerful risk factor in the disease’s progression. Two of the miRs, -21-5p and -214-3p, were additionally associated with an increased risk of kidney failure, while miR-192 was associated with the rate of eGFR decline and, along with miRs -141 and -200c, correlated with markers of epithelial–mesenchymal transition (EMT), vimentin and E-cadherin, establishing their roles in fibrosis. Unfortunately, these studies lacked appropriate non-IgAN kidney disease controls, making it impossible to draw conclusions about the specificity of the miRs for IgAN. We have recently demonstrated in frozen renal biopsy cores that four miRs, -150-5p, -146b-5p, -155-5p and -135a-5p, were differentially expressed in IgAN patients with the progressive form of the disease compared to those with stable IgAN and correlated with clinical parameters associated with progression including eGFR, proteinuria, and MEST-C scores [50]. Moreover, each miR increased the discrimination score of the International IgAN Prediction Tool, although due to the small number of samples the results did not reach statistical significance. Of the four miRs, miR-150-5p exhibited the largest amplitude of expression between cohorts and displayed the best discrimination between IgAN progressors and IgAN non-progressors by ROC analysis (AUC: 0.8). However, miR-150 expression was similarly upregulated in kidneys with established fibrosis and low eGFR at time of biopsy, consistent with a more generic role in kidney fibrosis and disease progression [50].

Investigations performed on laser capture micro-dissected (LCMD) kidney biopsy tissue have been able to shed light on miR expression in specific compartments of the nephron, significant differential expression of which may be missed when analysing whole biopsy cores. In a comprehensive study investigating miR expression in glomeruli and proximal tubules across four kidney diseases including IgAN, Baker et al. [51] discovered that some miRs were exclusively expressed in glomeruli while others in proximal tubular cells. Surprisingly, only a few miRs expressed disease-specific differences in glomeruli, while none were found in proximal tubular cells. In IgAN compared to healthy subjects, two miRs were differentially expressed in glomeruli (miR-3182 and -486-5p), while eight miRs were differentially expressed in proximal tubules (miRs -451a, -486-3p, -486-5p (downregulated), miRs -125b-5p, -99a-5p, -1303, -1273g-3p (upregulated)). Interestingly, no miRs were differentially expressed between IgAN and diabetic nephropathy. A study, specifically investigating the expression of miRs in glomeruli exhibiting endothelial proliferation and hypercellularity, found that miR-223 was downregulated in glomeruli displaying this lesion compared to those that did not [52]. Further experimental investigations replicating this scenario in vitro demonstrated that patient-derived IgA could induce the release of IL-6 by mesangial cells. The addition of this mesangial cell ‘conditioned medium’ to glomerular endothelial cells resulted in a downregulation of miR-223 levels and induced proliferation, monocyte adhesion and expression of intracellular adhesion molecule 1 (ICAM-1). The same authors also investigated the role of miR-21 [49]. They found that, compared to healthy subjects, miR-21 was significantly increased in both glomeruli and the interstitium of IgAN kidneys. In vitro follow-up experiments exposing mesangial cells to IgA from either IgAN patients or healthy subjects revealed that greater levels of TGF-β and TNF-α were secreted in response to IgA from IgAN patients. Moreover, the addition of these cytokines to podocytes and proximal tubular cells (HK-2) also significantly increased levels of miR-21, an effect which could be abrogated in the presence of cytokine-neutralising antibodies. The fact that the inhibition of miR-21 was also able to reduce the expression of EMT markers and suppress the expression of the proliferation pathway regulator PTEN (phosphatase and tensin homolog) confirmed that this miR was also involved in fibrosis in IgAN. 

In related experiments, Guo et al. [53] found that the expression of the miR-200b,c/429 cluster was downregulated in IgAN kidney tissue, as well as in cultured podocytes generated from IgAN tissue and in HK-2 proximal tubular cells compared with matched controls. Overexpression of this miR cluster in IgAN-podocytes and HK-2 cells reduced the release of inflammatory cytokines MCP-1, IL-6 and RANTES. This is likely to have occurred as a result of miR-200b,c/429 targeting the 3’UTR region of TWEAK (TNF-like weak inducer of apoptosis), thereby inhibiting NF-KB pathway activation and supressing inflammation [53]. 

In a separate study, high throughput RNA sequencing indicated that the miR-29 cluster was similarly suppressed in IgAN kidney tissue [45]. However, validation by RT-PCR showed that only miR-29b-3p of this cluster was significantly downregulated in IgAN compared to healthy subjects [54]. Following in silico identification of putative targets, and their confirmation by luciferase assays and Western blotting in HEK293T cells, it was shown that miR-29b-3p directly targeted CDK6, a p65 kinase responsible for the phosphorylation of p65 in NF-KB transcription factor signalling. The increased NF-KB signalling promoted by p65 phosphorylation resulted in increased cytokine secretion replicating the inflammatory conditions found in the IgAN kidney.

**Figure 3:**
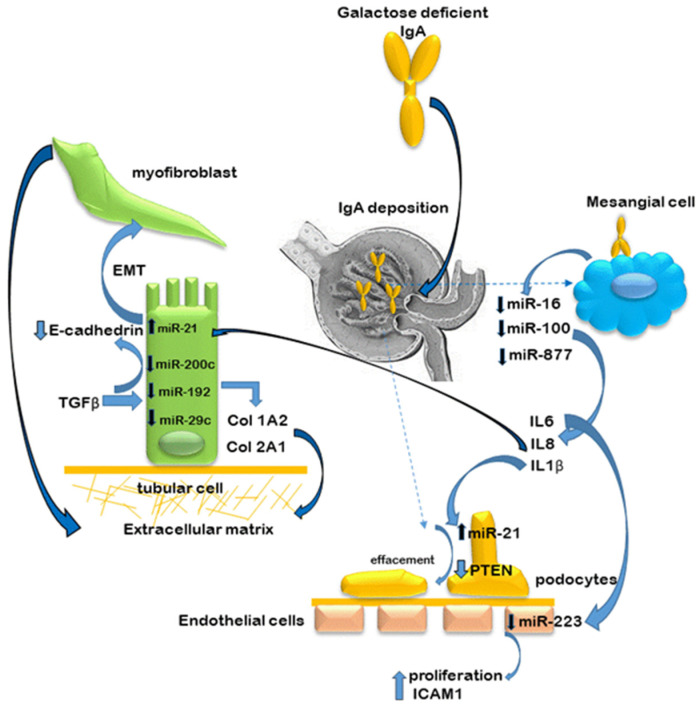
Intrarenal miR interactions. This diagram illustrates the result of dysregulated miR interactions on the cells of the nephron leading to inflammation and fibrosis. For clarity, only some of the major interactions have been described. IgA deposition downregulates miRs -16, -100 and -877 in mesangial cells, which induces the generation of cytokines IL-6, IL-8 and IL-1β. Subsequently, IL-8 upregulates expression of miR-21 in tubular cells and downregulates E-cadherin as part of the process of epithelial–mesenchymal transition (EMT)—a hallmark of fibrosis. IL-1β upregulates miR-21 expression in podocytes reducing phosphatase and tensin homologue (PTEN) expression and inducing podocyte effacement. IL-6 downregulates miR-223 expression in endothelial cells causing proliferation and expression of intracellular adhesion molecule (ICAM1). In the tubular cells, TGB-β downregulates expression of miRs-200c, -192 and -29c which induces EMT by reducing E-cadherin and increasing collagen deposition.

In experimental attempts to reproduce the inflammatory effects of IgA deposition in the glomeruli in IgAN, cultured mesangial cells have been stimulated with secretory IgA. These experiments demonstrated an overproduction of proinflammatory cytokines IL-6, IL-8 and IL-1β compared to non-stimulated cells. Subsequent in silico analysis revealed that these cytokines were targets of miRs -16, -100-3p and -877-3p, respectively. The in silico data were subsequently validated by RT-PCR and by transfection experiments using mimics and antagomiRs [55,56], and confirmed that IL-8 was a direct target of miR-100-3p while IL-1β was a direct target of miR-877-3p. Intrarenal miR interactions between the cells of the nephron are illustrated in Figure 3.

### 5.2. Extrarenal miR Expression

The fact that up to 60% of kidneys transplanted into IgAN patients develop glomerular mesangial IgAN deposits [57], while kidneys inadvertently transplanted from IgAN patients into those with non-IgAN CKD are cleared of their IgA deposits [58], has led to the concept that IgAN is a systemic disease, with kidney injury being part of the collateral damage. Therefore, searching for dysregulated gene expression beyond the kidney as a key to understanding its pathogenesis is well founded. 

### 5.3. Peripheral Blood Mononuclear Cells

Using microarray analysis of miRs expressed in circulating peripheral blood mononuclear cells (PBMC), Serino et al. discovered that 37 miRs were dysregulated in seven IgAN patients compared to healthy subjects [59]. Following validation, seven of the dysregulated miRs, -148b, -188-5p, -361-3p, -886-3p, -let-7b and -let7-d, were found to strongly associate with IgAN. Subsequent in silico analysis indicated that two of these miRs, -148 and -let-7b, targeted core 1 β1,3 galactosyltransferase (C1GalT1) and N-acetylgalactosyltransferase 2 (GALNT2), respectively, enzymes involved in the *O*-glycosylation of the IgA1 hinge region. Validation by RT-PCR revealed that the expression of both enzymes was lower in the PBMC of IgAN patients compared to healthy subjects. The regulatory effects of miR-148b and let-7b on these enzymes were confirmed using enforced expression/suppression transfection experiments using mimics and antagomiRs. Importantly, a positive correlation was found between miR-148b and the serum levels of poorly *O*-galactosylated IgA1. Of relevance to these findings, within the promoter region of C1GalT1 is a single nucleotide polymorphism (SNP) (rs 1047763) which is associated with the production of poorly *O*-galactosylated IgA1 [60]. Moreover, the position of the SNP coincides with the target region of miR-148b, thus providing a potential mechanism of action for this miR in the regulation of IgA1 *O*-glycosylation. MiR-148b has also been reported to negatively regulate the proximal tubular scavenger receptor megalin in IgAN, and may therefore impact on the proximal tubule’s ability to handle filtered IgA containing immune complexes [61].

More recently, two high throughput RNA sequencing studies on PBMC [62,63] from Chinese cohorts have been performed. One study discovered 44 differentially expressed miRs, 41 of which were novel to the pantheon of miRs associated with IgAN [60]. Ranked highest among these was miR-6843-3p. The second study revealed that miR-98-5p was highly differentially expressed in IgAN patients compared to healthy subjects. Enforced overexpression of miR-98 was able to reduce the expression of its target CCL3 (chemokine ligand 3) and concurrently increase IL-6 expression while reducing that of C1GalT1, suggesting that miR-98 may also be involved in the regulation of IgA1 *O*-galactosylation [63]. 

### 5.4. B Cells

PBMC are, of course, composed of a number of cell types, each with their specific functions. Experiments specifically performed on B cells in a Chinese IgAN cohort [64] demonstrated an upregulated expression of miR-347b compared to healthy subjects. Replicating this over-expression in normal B cells resulted in the suppression of Cosmc, the chaperone protein of C1GalT1, a reduction in the expression of PTEN at both mRNA and protein levels and increased B cell proliferation, which ultimately resulted in increased concentrations of poorly *O*-galactosylated IgA1. 

**Figure 4:**
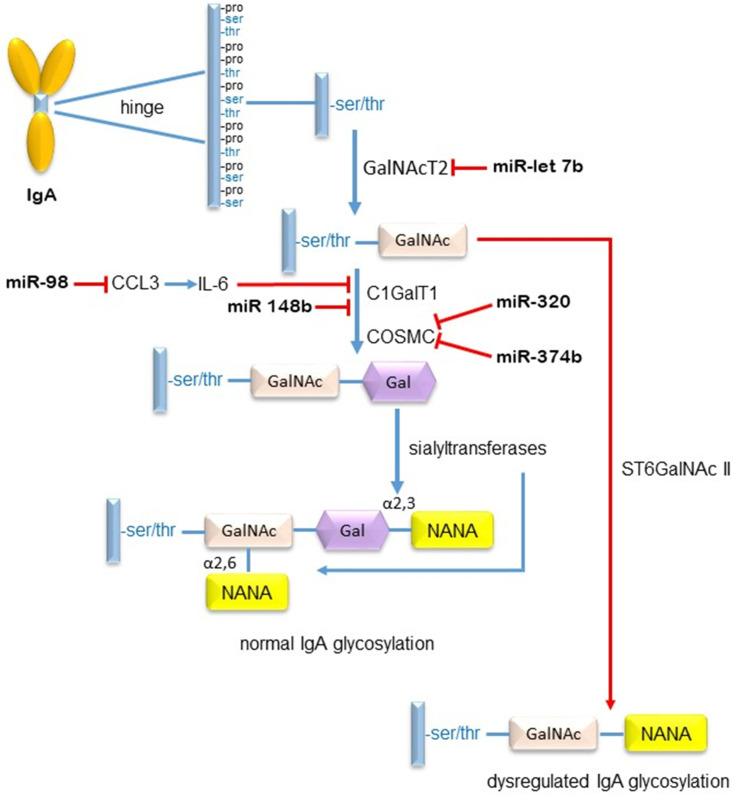
MicroRNAs which modulate IgA1 *O*-glycosylation. The process of IgA hinge region *O*-glycosylation is initiated by attachment of N-acetyl galactosamine (GalNAc) to a serine or threonine amino acid by the actions of N-acetyl-galactosamine transferase 2 (GalNAc T2). Galactose is sequentially attached by core 1 β,3-galactosyltransferase (C1GalT1) with the aid of its chaperone protein COSMC. N-acetyl neuraminic acid (NANA) can then be added to galactose by the actions of β-galactoside α-2,3 sialyl transferase 1 (ST3Gal1) for linkage in the α 2-3 orientation or to N-acetylgalactosamine by α-N-acetylgalactosaminide sialyl transferase 2 (ST6 GalNAc 2) for linkage in the α2-6 orientation. However, miR-let-7b can prevent the addition of GalNAc to its hydroxylated amino acid. MiR-148 and miR-98 can reduce the activity of C1GalT1, thereby reducing the binding of galactose to GalNAc. MiRs -320 and -374 can downregulate COSMC, reducing the ability of C1GalT1 to galactosylate N-acetyl galactosamine. Consequently, ST6GalNAc 2 can add NANA to the naked GalNAc sugar residue, preventing further extension of the sugar chain resulting in less *O*-galactosylation of IgA.

In similar experiments, having discovered that upregulated miR-320 expression in the renal tissue and urine of IgAN patients was accompanied by reduced expression of Cosmc, Li et al. overexpressed miR-320 in B cells and discovered that this had the effect of downregulating Cosmc expression as well as inducing B cell proliferation [65], again implicating this miR in the regulation of IgA1 *O*-glycosylation. Potential miR interactions resulting in the galactosylation of IgA are illustrated in Figure 4.

### 5.5. T Cells

IgAN is characterised by higher proportions of Th2, Tfh, Th17, Th22 and gamma delta (γδ) T cells with lower proportions of Th1 and regulatory T cells (Tregs) compared to healthy subjects [66]. Microarray analysis followed by validation in PBMC in a Chinese IgAN cohort revealed a lower expression of miR-155 in PBMC of IgAN than in healthy subjects. This was accompanied by lower expression of mRNA Cosmc and forkhead box P3 (*FOXP3*), a master regulator of the development and function of Tregs [67]. Flow cytometry revealed that these changes were associated with a change or ‘drift’ in T cell phenotype manifested as increased numbers of Th2 and Th17 cells with concurrent decreased Th1 and Tregs [67]. In a similar study, reduced numbers of Tregs were also observed in PBMC in IgAN, but this was accompanied by reciprocal increases in miRs -133a and -133b compared to healthy subjects—miRs known to target *FOXP3* [68]. 

Following analysis of PBMC miR expression by next generation sequencing, Xu et al. identified 22 differentially expressed miRs in IgAN patients compared to healthy subjects, of which miR-21 exhibited the highest expression and negatively correlated with putative targets Sprouty (*SPRY*) 1 and 2, and Fas ligand (*FASLG*). Follow-up studies in T cells showed that raised levels of miR-21 were detected in CD3+ T cells of IgAN patients, which negatively correlated with *SPRY1* expression and, importantly, induced Th17 cell polarisation, an effect which correlated with increased plasma IgA1 levels [69]. 

Despite the fact that most of these studies on peripheral blood cells were undertaken on restricted ethnic cohorts, they underscore the regulatory potential of miRs on IgA1 *O*-glycosylation, a process that is regarded as central to the generation of the pathogenic form of IgA1. 

### 5.6. Erythrocytes

Haematuria is a well-recognised clinical hallmark of IgAN, and when combined with proteinuria is a risk factor for future IgA progression. Mature erythrocytes are vehicles for a number of RNA species including miRs. Among the top 50 miRs expressed by erythrocytes, two thirds of them may have nephrotoxic potential in IgAN. It is thought that the compressive effects on erythrocytes as they cross the damaged glomerular basement membrane in IgAN may induce the production of erythrocyte-derived vesicles. These vesicles, which contain an array of miRs, are hypothesised to be capable of binding to and entering tubule cells as they pass down the nephron, releasing their miR payload in the process and thereby modulating the recipient cell transcriptome and cell phenotype [70]. 

## 6. Extracellular miRs

Since the discovery in 2008 that miRs also exist in a stable form outside of the cell [71,72], there has been a myriad of miR investigations in many different biological samples including blood (serum or plasma), urine, saliva, faeces, follicular fluid, synovial fluid, pancreatic juice, bile and gastric juice. Extracellular miRs are very stable and protected from degradation and denaturation by RNAses, high temperatures and variations in pH [73] owing to their containment within extracellular vesicles such as exosomes, microvesicles and apoptotic bodies, or by virtue of being complexed to proteins such as those of the AGO family [74] or high-density lipoproteins [75]. Which of the two modes of extracellular existence predominate in bodily fluids is an area of some debate. Some studies suggest that the majority of extracellular miRs are associated with AGO proteins [76] and that cells actively release functional miR-induced silencing complexes into the circulation [77]. However, other studies report that exosomal miRs are the major form, at least in serum and plasma [78]. It is entirely plausible that the protected state will depend on a number of parameters including the miR itself, its cellular source and the prevailing disease milieu. Interestingly, AGO-associated miRs within exosomes have also been reported [79].

Although some extracellular miRs may be by-products of cellular activity released following injury or death [80] and endure in the circulation due to the stability of the AGO complex, it is now generally recognised that extracellular miRs can also be selectively released in a regulated process, and function as intercellular and inter-organ modes of communication [81]. Once released from the source cell or tissue, they can be taken up by target cells either proximally or distally situated, resulting in the alteration of the target cells’ phenotype [82]. How miR vehicles deliver their cargo to specific distal sites remains to be fully determined. Evidence suggests that exosomal miRs can be taken up by endocytosis [83] or via interactions with connexins [84], integrins [84] and glycan pattern recognition [85], while vesicle-free, protein-bound miRs may be taken up by specific receptors [86].

### 6.1. Serum and Plasma

Further to their studies in PBMC [59], and based on the assumption that circulating extra-cellular miRs are derived from circulating cells, Serino et al. undertook a retrospective study looking for the expression of miRs-148b and let 7b in serum and extended their investigations to include East Asian patients [87]. While miR-let-7b expression in serum reflected that in PBMC, expression levels of miR-148b were significantly lower in serum. However, the expression of both miRs was higher in Caucasians than in East Asians. Despite these differences, the combined two miR signature was able to discriminate IgAN patients from healthy subjects in both the Caucasian and East Asian cohorts, suggesting that these miRs may have a universal role in the pathogenesis of IgAN. 

Wu et al. [88] found a signature of four miRs, -148-3p, -150-5p, -20a-5p and -425-3p, differentially upregulated in the plasma of IgAN patients compared to healthy subjects. Receiver operating statistic (ROC) curves revealed areas under the curve (AUC) of 0.8 and 0.76 for the training and testing stages, respectively. Moreover, the expression of the miR signature was higher in the I-II subgroup of the IgAN Lee’s grading system [89] than the III-V subgroup, suggesting that the miR signature was able to distinguish IgAN from healthy subjects at an early stage of disease. Bioinformatic analysis revealed that these miRs had potential functional links with TGF- signalling pathways and fibrosis. Unfortunately, as no non-IgAN kidney disease controls were included in the study, it was not possible to ascertain whether this miR signature was specific for IgAN.

As previous studies had shown that miR-29 was involved in kidney fibrosis as a result of modulating TGF-β1/Smad4 signalling, Hu et al. [90] aimed to establish whether plasma levels reflected this dysregulated expression in order to utilise it as a potential biomarker for monitoring responses to treatment and severity of disease. They found that miR-29a expression in IgAN was significantly lower than that in healthy subjects. Moreover, plasma miR-29a concentrations positively correlated with eGFR and negatively with proteinuria and serum creatinine. Levels also negatively correlated with both the Lee and Oxford histological scores [89,91]. Crucially, Kaplan–Meier curves indicated that patients with raised plasma levels of miR-29a had better kidney function and an improved response to treatment, suggesting that this miR could be used as a prognostic biomarker in a clinical setting. Unfortunately, no non-IgAN kidney disease controls were included in this study and so it was not possible to ascribe their observations specifically to IgAN. 

In a similar vein, Fan et al. [92] sought to establish a potential association between the intrarenal expression of miR-192 with serum and urinary exosome levels in IgAN patients compared to healthy subjects. They confirmed that intrarenal miR-192 expression correlated with serum levels, eGFR, and with severe interstitial lesions, tubular atrophy and interstitial inflammation. However, no such correlations were found for this miR in urinary exosomes. 

### 6.2. Urine

Urine is one of the least invasive of available biological samples and is therefore a highly convenient tissue for biomarker discovery studies. Not surprisingly, the number of miR analysis studies performed in urine is rising exponentially.

Numerous studies have investigated urinary miRs in IgAN, either exclusively or in multi-kidney disease cohorts. Early investigations concentrated on specific miRs previously reported to be dysregulated in intrarenal studies in IgAN. These studies showed that miRs -200a, -200b and -429 were downregulated in the urine in IgAN and that downregulation correlated with disease severity and rate of progression [93], while miRs-146a and -155 were elevated in the urine in IgAN and correlated with clinical and histological severity [47]. In addition, urinary miRs-29b and -29c have been reported as being down-regulated in IgAN and correlated with the level of proteinuria and kidney function [94]. Furthermore, it has been suggested that urinary excretion of miRs-34a, -205, -155 and -21 could distinguish IgAN from healthy subjects, while miRs -21 and -205 could distinguish those IgAN patients with tubulointerstitial damage [95]. More recently, it has been shown that miR-152-5p levels are higher in the urine in IgAN compared to other forms of CKD, and that miR-152-5p levels also correlate with the level of proteinuria and degree of pathological damage [96]. 

The ability to screen the urine miRnome using microarrays, and high throughput RNA sequencing, currently the most effective method of ‘mining the miRNome’, has extended the evidence base for urinary miR composition in IgAN. Wang et al. [97] used microarray analysis to investigate miR expression in the urine sediments of a Chinese IgAN cohort compared to a non-IgAN disease cohort that included membranous nephropathy and minimal change disease, as well as healthy subjects. Following screening and validation, one miR, -3613-3p, was found to be significantly downregulated in IgAN compared to non-IgAN CKD and healthy subjects, while miR-4668 was significantly differentially expressed in IgAN compared to healthy subjects. Levels of both miRs correlated with Lee Classification I-II and III subgroups, as well as 24hr proteinuria, and eGFR. In addition, both miRs -3613 and -4668 were downregulated in kidneys displaying no segmental sclerosis (S0 of the Oxford classification system) compared to the S1 lesion. Szeto et al. [98] performed a similar study, employing the nanostring microarray platform. The group found a miR signature (miRs -204, -431, -555 (downregulated) and miR-150 (upregulated)) which was significantly differentially expressed in the urine sediments of IgAN patients compared to healthy subjects. One of the miRs, miR-204, had a discriminatory AUC by ROC analysis of 0.976. However, with the exception of miR-555, which exhibited a moderate association with duration of disease and baseline eGFR, none of the miRs significantly correlated with any clinical parameters. Unfortunately, this study did not include non-IgAN kidney disease controls, making it impossible to assess the specificity for IgAN. 

A separate miR chip assay on urine sediments reported that miRs -25-3p, -144-3p and -486-5p all exhibited good specificity and sensitivity for IgAN diagnosis by ROC analysis, with miR-486-5p exhibiting the largest AUC at 0.935 [99]. Moreover, miRs-144-3p and -486-5 were also significantly increased in vesicles extracted from the urinary supernatants following sedimentation [99]. 

By contrast, a study of urinary exosomes [100], rather than sediment, and employing high throughput RNA sequencing found that miRs -215 and -378i were upregulated and miRs -29c and -205-5p were downregulated in IgAN compared to healthy subjects. However, no further validation of these data was undertaken. Yu et al. [101] in another study of urinary exosomes from patients with CKD, including IgAN and healthy subjects, found that miR-200b exhibited the lowest expression in the CKD group compared to healthy subjects. Interestingly, when the exosomes were sorted into those derived from proximal tubular cells (CD13+) and those derived from other tubular cells (CD13-), they discovered that miR-200b suppression was more strongly associated with the non-proximal tubular cell population [102]. 

Whether urinary miR expression reflects that of kidney tissue expression is still a matter for debate. Some studies show good correlation [102], while others report the opposite. For example, miRs -150-5p and -223 -3p appear to be abundantly expressed in urine [102], but are downregulated in kidney tissue [45]. One explanation to account for these differences may be the ability of erythrocytes to ‘load up’ with miRs. In support of this, Duan et al. [99] found that miR levels were highest when erythrocytes had not been removed from the urine sediment, perhaps not surprising, since erythrocytes bear the highest levels of miRs of any cell type found in the urine. As no difference in circulating erythrocyte miR levels was detected between IgAN patients and healthy subjects, it was surmised that loading must have occurred within the kidney itself [99]. 

By way of an overarching summary, although not exclusive to IgAN patients, Konta et al. [102] concluded that urinary miR concentrations increase with the severity of kidney disease, their levels generally correlate with proteinuria and glomerulosclerosis and, more importantly, different diseases exhibit different patterns of miR expression. 

Despite the non-invasive, freely available nature of urine as a medium for biomarker discovery, it is clear that the urinary miR composition differs between studies depending on the whether the miRs were investigated in sediment or exosomes, using microarrays, chips or high throughput RNA sequencing and, importantly, on the stage of disease being investigated. Most of the studies described here were undertaken in Chinese cohorts, and currently there are still very few studies reporting miR expression profiles in Caucasians.

## 7. MiRs and Genetic Susceptibility to IgAN

The advent of genome-wide association studies (GWAS) has identified numerous IgAN disease-associated risk loci which have shed light on potential genes, mechanisms and signalling pathways associated with the susceptibility for the development of IgAN. Some of these have also been shown to associate with potential miR target sites. As mentioned earlier in this review, a target site for miR-148b has been detected in SNP rs1047763 within the gene *C1GalT1* [60], which encodes for a key enzyme responsible for *O*-galactosylation of IgA1.

A Chinese study investigating SNPs in the 3’UTR regions of four genes (*TNS3*, *PHLDB1*, *NTN4*, *GNG2*) as potential risk loci for IgAN [103] found that they all exhibited associations with IgAN. However, the most noteworthy correlations were found with the homozygotic genotype rs136270 A/A within the Netrin-4 (*NTN4*) 3’UTR, which was not only associated with a higher risk of IgAN [103], but also contained a potential target site for miR-483-5p.

A more recent Chinese GWAS identified four SNPs within the *FCRL3* (Fc receptor-like 3) gene which were significantly associated with IgAN susceptibility. The rs11264794 SNP correlated with kidney outcome and contained a potential target site for miR-183-5p. The A allele was found to be protective for IgAN and correlated with higher gene expression, while the minor allele resulted in reduced miR-183 binding and an increased susceptibility for IgAN [104]. 

MiR-146a is recognised as a regulator of the immune response and is known to be upregulated in patients with IgAN [47]. Within the miR-146a precursor is a functional SNP, rs2910164 C>G, which is known to affect the expression of mature miR-146a [105]. Interestingly, echoing the prevalence of IgAN, the frequency of this SNP differs between subjects of East Asian origin and Europeans, with a higher C:G ratio in Chinese compared to Europeans (0.646:0.354 in Chinese, 0.325:0.675 in Europeans). This SNP has been shown to be associated with the susceptibility to, and severity of, childhood IgAN [105]. In contrast, no such associations were observed in adult IgAN patients with the exception of a correlation with age of IgAN onset, suggesting that this SNP not only plays a role in miR-146a expression but also in the development of IgAN in East Asian populations [106]. However, these studies still require validation. It is worth noting that miR-146a serves as a negative feedback regulator of the innate immune system, modulating the activation of Toll-like receptor (TLR) pathways via nuclear factor KB [107,108]. The activation of TLR pathways is strictly regulated to prevent the hyperactivation of the innate immune system response and excessive inflammation. This suggests that the intra-renal action of miR-146a may be part of a final common pathway for interstitial scarring in inflammatory kidney diseases and not specific to IgAN. 

## 8. miRs as Biomarkers in IgAN

As might be imagined, with the growing number of studies reporting miR dysregulation in IgAN, a number of investigators have evaluated these changes as possible biomarkers in IgAN. Biomarkers can be invaluable tools in clinical practice, where they can guide diagnosis, prognostication, treatment selection, and the assessment of treatment response. It is, however, critical that when evaluating a novel analyte as a clinical relevant biomarker that the following factors are considered:

(1) **Biologically plausible**, with a clear role in mediating the disease of interest;
(2) **Stable at room temperature**, to minimise degradation between collection and processing;
(3) **Readily measurable** with available and affordable techniques;
(4) **Minimally invasive** to collect;
(5) **Generalisable** across populations;
(6) **Validated** prospectively and independently.

If a biomarker is to be used for diagnosis, it must also be:

(7) **Sensitive**; its presence should reliably diagnose the disease;
(8) **Specific**; its absence should reliably exclude the disease.

As reviewed above, a number of miRs have been identified in kidney, urine and blood that have been reported as candidate biomarkers in IgAN. A number of these do fulfil some of the criteria listed above; they modulate relevant disease pathways (biologically plausible) [53,54], most are stable at room temperature [71], can be measured using accessible technology, RT-PCR [109], and are differentially expressed in the blood and urine of IgAN patients (minimally invasive). However, to date no single/panel of miR biomarkers has demonstrated generalisability across populations, or within a population across different sites. Most critically, none have been independently validated and systematically shown to add value above and beyond biomarkers currently routinely used in IgAN:

(1) **Immunofluorescencestaining** of kidney tissue, which remains the only tool for diagnosis [110].
(2) **Light microscopy analysis(MEST-C score)** of kidney tissue for prognostication [91,111,112].
(3) **Proteinuria** for prognostication and monitoring response to treatment [113,114,115,116].
(4) **Creatinine-based eGFR** for prognostication and monitoring response to treatment [117,118,119,120,121,122].
(5) **Blood pressure (BP)** for prognostication and monitoring response to treatment [117].

These conventional biomarkers have been extensively validated across ethnically diverse populations, and are the basis for the International IgAN Risk Prediction Tool, which calculates the percentage risk for disease progression, for up to five years from the time of kidney biopsy [123]. Furthermore, the modification of these biomarkers, such as proteinuria and BP, improves outcomes in IgAN [123]. These features, in addition to their accessibility and affordability, make this set of biomarkers currently invaluable for prognostication and monitoring response to treatment. Despite these strengths, conventional biomarkers have two major weaknesses: diagnosis is still reliant upon an invasive kidney biopsy, and traditional biomarkers do not guide treatment selection, and are unlikely to do so as new treatment options emerge. The future of IgAN management is likely to be reliant on targeted therapeutics, and traditional biomarkers do not yield sufficient insights into pathway activation to guide such treatments. Any miR biomarker that reliably addresses these issues, whilst at least matching the utility of traditional biomarkers, is likely to add significant value to clinical practice. 

At present, no study of miRs in IgAN provides sufficient data to justify their use as a biomarker in IgAN. Most studies have been performed in a single centre using small patient cohorts, and none have been validated prospectively and independently. This does not mean to say that this will not happen in the future, but at present the literature is full of miR biomarker discovery studies whose findings are yet to be validated. 

Measurement of the serum levels of miR-148b and let-7-b has been proposed as a non-invasive diagnostic test for IgAN based on limited data from a multi-ethnic cohort of 145 East-Asians and Caucasians. However, these findings have yet to be prospectively independently validated, and together yield relatively low diagnostic sensitivity (64%) and specificity (74%) using ROC analysis. 

The levels of a number of miRs have been associated with worse clinical outcomes in IgAN; however, this likely reflects the non-specific association of these miR clusters with generic inflammatory and fibrotic cascades [48,93,124]. Importantly, until recently none of these studies had formally evaluated the additive value of miR measurement above those biomarkers that contribute to the International IgAN Risk Prediction Tool, the current gold standard for risk prediction in IgAN. The first study to do this reported a small improvement in risk prediction by the addition of intrarenal levels of 4 miRs (-150-5p, -155-5p, -146b-5p and -135a-5p) to the IgAN Prediction Tool, with miR-150-5p providing the strongest discrimination between IgAN patients with progressive and non-progressive disease [50]. This study was, however, limited in size and has not yet been validated, but does suggest that the measurement of miRs may in the future improve the current approach to risk prediction in IgAN. 

In summary, although there are an increasing number of publications reporting the dysregulation of miR expression in IgAN, none have yet reached the evidence threshold required to justify their transition from interesting observation to clinically relevant biomarker.

## 9. RNAi Therapeutics in IgAN

The current treatment of IgAN is limited by an incomplete understanding of the mechanisms that drive disease initiation and progression. Therapeutic options are focused on goal-directed supportive care, which includes BP control, renin–angiotensin system (RAS) inhibition, lifestyle modification and cardiovascular risk management [125]. Despite being considered supportive, these interventions are safe and effective. RAS inhibition reduces proteinuria, controls BP and reduces the rate of decline of eGFR in IgAN [126]. However, the long-term outcome of the STOP-IgAN clinical trial, which demonstrated no added benefit of systemic immunosuppression over supportive care, found that despite optimal supportive care, 50% of the cohort reached a composite of death, ESKD, or a decline of over 40% in eGFR over a median follow-up of 7.4 years. There is a clear unmet need for new therapeutic approaches in IgAN which will hopefully be addressed as the pathophysiological pathways operating in IgAN are more precisely defined. 

In particular, it is hoped that a more complete understanding of the regulation of gene transcription and post transcriptional processes by miRs may offer novel therapeutic targets. Furthermore, by virtue of their structure, ncRNAs are excellent candidates as drugs themselves, capable of delivering a highly targeted approach to therapy. They can be synthesised with relative ease and designed to silence selected transcripts critical to specific disease-causing biological pathways [127]. RNAi therapeutics offer the ability not only to release transcripts from endogenous suppression, thereby promoting protein synthesis, but also directly disrupt the translation of transcripts, thereby causing protein synthesis suppression [128]. RNAi therapeutics are already being evaluated in IgAN: 

**Cemdisiran** is a short oligonucleotide with a sequence complementary to the transcript of complement component C5. C5 is part of the terminal complement pathway and contributes to the formation of the membrane attack complex and the generation of the potent anaphylatoxin C5a. Complement activation is one of the key inflammatory pathways activated by mesangial IgA deposition and a significant contributor to glomerular injury in IgAN [129]. Cemdisiran is designed to downregulate the synthesis of C5 in hepatocytes through a mechanism identical to that employed by an endogenous miR, utilising the RISC complex to deplete C5 mRNA [130]. Cemdisiran is currently being evaluated in a phase II clinical trial in IgAN (NCT03841448).

**Ionis-Fb-L_rx_** is a short length antisense oligonucleotide with a sequence complementary to the transcript of complement factor B (CfB), a component of the alternative complement pathway [131]. Analogous to Cemdisiran, Ionis-Fb-L_rx_ binds the CfB transcript, preventing its translation in hepatocytes and suppressing circulating levels of Cfb. It is currently being evaluated in a phase II trial in IgAN (NCT04014335).

The development of RNAi therapeutics is not without its challenges. Their short nucleotide lengths confer a high degree of promiscuity, with a single oligonucleotide capable of modulating hundreds of transcripts [132]. This can result in an array of off-target effects, unless carefully designed mechanisms are employed to deliver an RNAi therapeutic to specific tissues of interest. Despite adding a layer of complexity to drug design, this is feasible. Both Cemdisiran and Ionis-Fb-L_rx_ are conjugated to *N*-acetylgalactosamine, which binds with high affinity to the asialoglycoprotein receptor expressed almost exclusively by hepatocytes. This directs these RNAi therapeutics specifically to the hepatocyte, the principal site of C5 and factor B synthesis [133,134,135]. Such approaches, while still in their infancy, offer an insight into the potential future contribution of RNAi therapeutics to the field of precision medicine generally, and IgAN specifically.

## 10. In Summary

It is clear that there is an increasing appreciation of the potential role that miRs and other ncRNAs play in modulating IgAN pathogenesis, and that miRs have great potential to deliver both transformative biomarkers to help guide the management of patients with IgAN and offer a new approach to the delivery of targeted therapy. However, despite the many publications to date describing miR dysregulation in IgAN, the existing data must at present be considered hypothesis generating. Going forward, there needs to be a concerted effort to consolidate our understanding with well designed, cross-population validation studies to rigorously evaluate the role of miRs in IgAN pathogenesis and as future clinically meaningful biomarkers. 

## Figures and Tables

**Table 1:** The different types of non-coding RNAs, divided according to their function: housekeeping and regulation [1].

RNA Type	Abbreviation	Full Name	Size (nt)
	rRNA	ribosomal RNA	120–4500
	tRNA	transfer RNA	76–90
	snRNA	small nuclear RNA	100–300
Housekeeping ncRNA	snoRNA	small nucleolar RNA	60–400
	TERC	telomerase RNA	/
	tRF	tRNA-derived fragments	16–28
	tiRNA	tRNA halves	29–50
	miR	microRNA	21–23
	siRNA	small interfering RNA	20–25
	piRNA	piwi-intreracting RNA	26–32
Regulatory ncRNA	eRNA	enhancer RNA	50–2000
	lncRNA	long noncoding RNA	>200
	circRNA	circular RNA	100–10,000
	Y RNA	Y RNA	/
